# Implementation of a lifestyle intervention for type 2 diabetes prevention in Dutch primary care: opportunities for intervention delivery

**DOI:** 10.1186/1471-2296-13-79

**Published:** 2012-08-08

**Authors:** Paulina WA Vermunt, Ivon EJ Milder, Frits Wielaard, Caroline A Baan, Jos DM Schelfhout, Gert P Westert, Hans AM van Oers

**Affiliations:** 1 Scientific Centre for Transformation in Care and Welfare (Tranzo), University of Tilburg, Warandelaan 2, 5037, AB, Tilburg, the Netherlands; 2 Centre for Prevention and Health Services Research, National Institute for Public Health and the Environment, Antonie van Leeuwenhoeklaan 9, 3721, MA, Bilthoven, the Netherlands; 3 Association of primary care practices ‘De Ondernemende Huisarts’ (DOH), P.O. Box 704, 5600, AS, Eindhoven, the Netherlands; 4 Scientific Institute for Quality of Healthcare (IQ Healthcare), Radboud University Nijmegen Medical Centre, P.O. Box 9101, 6500, HB, Nijmegen, the Netherlands; 5 Centre for Public Health Forecasting, National Institute for Public Health and the Environment, Antonie van Leeuwenhoeklaan 9, 3721, MA, Bilthoven, the Netherlands

**Keywords:** Type 2 diabetes, Primary care, Lifestyle intervention, Implementation

## Abstract

**Background:**

As in clinical practice resources may be limited compared to experimental settings, translation of evidence-based lifestyle interventions into daily life settings is challenging. In this study we therefore evaluated the implementation of the APHRODITE lifestyle intervention for the prevention of type 2 diabetes in Dutch primary care. Based on this evaluation we discuss opportunities for refining intervention delivery.

**Methods:**

A 2.5-year intervention was performed in 14 general practices in the Netherlands among individuals at high risk for type 2 diabetes (FINDRISC-score ≥ 13) (n = 479) and was compared to usual care (n = 446). Intervention consisted of individual lifestyle counselling by nurse practitioners (n = 24) and GPs (n = 48) and group-consultations. Drop-out and attendance were registered during the programme. After the intervention, satisfaction with the programme and perceived implementation barriers were assessed with questionnaires.

**Results:**

Drop-out was modest (intervention: 14.6 %; usual care: 13.2 %) and attendance at individual consultations was high (intervention: 80-97 %; usual care: 86-94 %). Providers were confident about diabetes prevention by lifestyle intervention in primary care. Participants were more satisfied with counselling from nurse practitioners than from GPs. A major part of the GPs reported low self-efficacy regarding dietary guidance. Lack of counselling time (60 %), participant motivation (12 %), and financial reimbursement (11 %) were regarded by providers as important barriers for intervention implementation.

**Conclusions:**

High participant compliance and a positive attitude of providers make primary care a suitable setting for diabetes prevention by lifestyle counselling. Results support a role for the nurse practitioner as the key player in guiding lifestyle modification. Further research is needed on strategies that could increase cost-effectiveness, such as more stringent criteria for participant inclusion, group-counselling, more tailor-made counselling and integration of screening and / or interventions for different disorders.

## Background

Type 2 diabetes is a serious illness, associated with severe complications and increased mortality [1]. Global prevalence is estimated to rise to 438 million in two decades, increasing the worldwide burden posed by this disease [1]. Studies in experimental settings have however shown that type 2 diabetes incidence and risk can greatly be reduced by lifestyle counselling in high risk individuals [2]. In the Netherlands, the ‘Study on Lifestyle intervention and Impaired glucose tolerance Maastricht’ (SLIM) demonstrated a reduction of diabetes incidence of nearly 60 % in 4 years in individuals that completed a three-year lifestyle intervention [3]. The beneficial effects of these behavioural interventions can be maintained long after counseling is stopped [4,5].

As in clinical practice resources may be limited compared to experimental settings, implementation of successful lifestyle interventions into daily life settings is challenging [6,7]. It is therefore important to health policy makers to gain insight into factors influencing translation of evidence-based programmes into ‘the real world’ [7,8]. A better understanding of programme implementation may furthermore reveal opportunities for refining intervention delivery [7,8]. However, despite growing evidence on effectiveness of diabetes prevention in routine health care [9-13], evaluation of intervention implementation remains limited [7].

The ‘Active Prevention in High Risk individuals of Diabetes Type 2 in and around Eindhoven’ (APHRODITE) study investigates type 2 diabetes prevention in Dutch primary care. General practice was chosen for implementation, because in the Netherlands patients consider the GP trustworthy [14] and ~99 % of inhabitants are registered with a practice [15]. Furthermore, Dutch GPs attach importance to primary prevention of chronic diseases and the majority of practices have a nurse practitioner to support preventive activities [16]. In this article we evaluate participant compliance with the intervention, attitudes and expertise of providers, satisfaction with the structure and intensity of the intervention and perceived implementation barriers of providers. Based on these insights we discuss opportunities for refining intervention delivery on different healthcare levels [8].

## Methods

Participants were recruited in January 2008 by 48 GPs and 24 nurse practitioners from an association of 14 primary care practices in the Netherlands. A Dutch translation of the Finnish FINDRISC [17] was sent to GP patients aged ≥40 and ≤70 years (n = 16032). The FINDRISC was validated in three Dutch cohorts and was found to be a reasonably good predictor of incident diabetes in the Netherlands [18]. All individuals with a score ≥13 points (n = 1533) were invited to participate in the intervention. Randomization was performed on the level of the individual. In total, 479 individuals were allocated to the intervention group and 446 individuals to the usual care group. Details of participant recruitment and intervention reach were described previously [19].

### Intervention protocol

The APHRODITE intervention was based on the transtheoretical model [20]. Stage transition was supported by using behavioural change techniques to influence motivation (motivational interviewing, decisional balance), action (goal setting, action planning, barrier identification) and maintenance (relapse prevention) [21,22]. Research objectives focused on weight loss, increasing the amount of physical activity and improving dietary composition. Clinical and lifestyle measurements were performed at baseline and after 6, 18 and 30 months. Details of participant measurement taking were described previously [23].

To ascertain regular contact with health care providers, 11 consultations of 20 minutes were scheduled over 2.5 years with alternately the nurse practitioner and the GP. To decrease workload for providers and stimulate contact between participants, five group-meetings of 1 hour were organised by dieticians and physiotherapists to provide more detailed information on diet and exercise. Moreover, all individuals were invited for a 1-hour personal consultation with the dietician. Participants were invited for the group-meetings in their own town directly by the dietician or physiotherapist. Participants could call the dieticians’ or physiotherapists’ office to join another meeting if the day or time didn’t suit them. Details of the planning of the intervention and content of the group-consultations are shown in Table 1.

**Table 1:** Planning of the APHRODITE intervention and content of the group-consultations

**Time**	**GP**	**NP**	**Group-consultation**	**dietician**
Baseline	Admission			
Baseline		Admission		
1 month			*Topics:* nutrition components; calories and fat	
1 month			*Topics:* carbohydrates; sugar; sweeteners	
2 months				Consultation
3 months		Follow-up		
6 months		Follow-up		
9 months	Follow-up			
9 months			*Topic:* exercise in relation to sugar metabolism	
12 months		Follow-up		
15 months	Follow-up			
15 months			*Topics:* food package labels; nutrition logo’s; fibres	
18 months		Follow-up		
21 months	Follow-up			
21 months			*Topics:* food packages; food game to recall information	
24 months		Follow-up		
27 months	Conclusion			
30 months		Conclusion		

The programme was free of charge for all participants. Providers received financial reimbursement for all consultations with their participants according to Dutch payment standards. The intervention was registered with the Dutch Trial Register (NTR1082). The Medical Ethical Review Committee of the Catharina Hospital in Eindhoven gave ethical approval to the study (M07-1705). All participants gave informed consent for participation.

### Usual care

During the admission interview, participants in the usual care group received oral and written information about type 2 diabetes and a healthy lifestyle. The nurse practitioner was visited only for measurements (10 minutes) at baseline and after 6, 18 and 30 months. Apart from the admission interview participants did not have study-related encounters with the GP.

### Training of GPs and nurse practitioners

Before the start of the study, all nurse practitioners and GPs received a two-evening training on the theoretical framework of the intervention and its translation into practice. In addition, all nurse practitioners received a five-evening course in motivational interviewing (MI) [24]. During the project, two return-meetings were organised with the GPs (once a year) and four with the nurse practitioners (every half a year). All nurse practitioners in our study were certified and had previously obtained a degree on higher vocational education level.

### Drop-out and attendance rates

A list of individuals ending participation was kept by the project assistant. Presence was registered by the GP or nurse practitioner (individual consultations) or by the dietician or physiotherapist (group-consultations). Attendance rates were calculated based on all persons participating at the particular time-point, excluding drop-outs (intervention: N = 70 (14.6 %); usual care: N = 59 (13.2 %) and individuals diagnosed with diabetes (intervention: N = 41 (10.0 %); usual care: N = 46 (11.9 %)). One practice was left out of attendance rate calculations as presence at individual consultations was not accurately registered.

### Participant and provider questionnaires

Questionnaires were developed and reviewed by an expert panel of epidemiologists, GPs and nurse practitioners. Provider questionnaires were filled out within one month after finishing the project. Response to the questionnaires was 80 % within GPs and 100 % within nurse practitioners. Participant questionnaires (intervention group) were filled out during the 30-month data collection. Response to this questionnaire was 84 %. As follow-up ended as soon as persons were diagnosed with diabetes or withdrew from the study, no records were available from drop-outs (N = 70 (14.6 %)) and individuals with diabetes (N = 41 (10.0 %)).

Confidence of professionals in diabetes prevention in primary care, confidence in diabetes prevention by lifestyle intervention and chance of success of diabetes prevention in primary care were assessed on 5-point Likert scales*.* Satisfaction of professionals with individual consultations was assessed on a 1 to 10 scale, questioning: ‘how much pleasure did you experience in consultations with intervention group participants?’. For analysis, 1–5 was categorised as ‘low’, 6–7 as ‘average’ and 8–10 as ‘high’.

Opinions of providers and participants on knowledge of providers were assessed with Yes/No-questions asking ‘do you think you have / your GP/nurse practitioner has enough knowledge on the following topics?’. Topics questioned were type 2 diabetes, complications of diabetes, a healthy weight, intakes of (saturated) fat and dietary fibre, benefits of exercise, and the amount of exercise needed. Participant satisfaction with lifestyle change guidance and providers perceptions of their suitability were assessed on 5-point Likert scales. Usefulness and desirability of the MI-course were also assessed on 5-point Likert scales.

Satisfaction of participants and professionals with the frequency and duration of consultations, usefulness of group-consultations, and opinions of participants on desirability of guided exercise programs and individual dietary counseling were assessed on 5-point Likert scales. Opinions of providers on desirability of guided exercise programs, on suitability of primary care for group-consultations, and on perceived organizational barriers for group-consultations were assessed with open questions.

### Sample size calculation

Sample size calculation was based on the main outcome diabetes incidence. Studies in experimental settings had shown reductions in diabetes incidence between study groups up to 58 % [2,3]. However as implementation of lifestyle interventions in real life settings is challenging [6,7], more modest differences in diabetes incidence were expected for this study. To detect small differences in diabetes incidence (Cohen’s conventional effect size of 0.1 [25]), with a power of 0.8, 393 individuals were needed in each arm. When a post-hoc correction for correlation on the nurse practitioner level was applied (variance 0.03), [26], this number changed to 405. As in total 925 individuals could be included, this allowed for a dropout rate of approximately 15 %, which was in line with others [2].

### Statistical analyses

Differences in baseline characteristics between the study groups were evaluated with independent samples’ t-tests or chi-square tests. Other differences between study groups and between subgroups of participants were evaluated using multilevel analysis (level 1: participant; level 2: nurse practitioner). As the clustering effects on the GP level (level 3) were neglectable after accounting for the effects of the nurse practitioner, the GP level was omitted. Changes in clinical outcomes of drop-outs were calculated from the last available measurement before withdrawal from the project. Analyses were performed using SPSS version 18.0 and SAS version 9.2. A p-value of <0.05 was considered significant.

## Results

Table 2 shows baseline characteristics of participants in both study groups. Mean total FINDRISC-score was higher in the usual care group than in the intervention group (p = 0.006). No other differences in baseline characteristics between the two study groups were found.

**Table 2:** Baseline characteristics of participants in both study groups

		**Intervention group**	**Usual care group**
N		479	446
Sex (% Male)		39.0	37.0
Age (years)		58.4 ± 7.4	58.1 ± 7.3
Education	% Low	52.8	50.7
	% Average	22.9	25.5
	% High	24.3	23.8
Smoking	% Yes	18.6	16.2
	% In the past	49.0	50.9
	% No	32.4	32.9
FINDRISC-score (points)		14.6 ± 2.0	14.9 ± 2.0
Body mass index (kg/m^2^)		29.0 ± 4.5	28.6 ± 4.2
FPG (mmol/l)		5.6 ± 0.6	5.6 ± 0.5
2 h PG (mmol/l)		6.0 ± 1.8	6.1 ± 1.9

Data are means ± SD unless otherwise indicated. * ; significant differences between groups as tested by either an independent samples t-test or a chi-square test. FPG = Fasting Plasma Glucose. 2 h PG = plasma glucose after two hours of oral glucose challenge. Low education = no education to lower vocational education. Average education = senior general secondary education to intermediate vocational education. High education = higher vocational education or university.

### Compliance of participants

After one year, 30 individuals in the intervention group (6.3 %) had dropped out of the programme compared to 33 in the usual care group (7.4 %). At the end of the programme (2.5 years), 70 participants (14.6 %) dropped out in the intervention group versus 59 (13.2 %) in the usual care group. Drop-out rates were comparable between groups (p = 0.517). Drop-outs on average were younger (56.2 years versus 58.6 years, p = 0.002) and less often had a partner (13.0 % versus 20.5 %, p = 0.03) than completers. Clinical outcome measures (body weight, blood glucose values) were comparable between drop-outs and completers. Reasons for withdrawal from the project were comparable between groups and included lack of time (24 %), disease (23 %), and moving (10 %).

Attendance of participants at individual consultations ranged from 80 % to 89 % (GP) and from 86 % to 97 % (nurse practitioner) in the intervention group and from 86 % to 94 % (nurse practitioner) in the usual care group (Table 3). In the intervention group, high attendance (75 % or more) was associated with larger reductions or smaller increases in blood glucose values (fasting plasma glucose: -0.17 versus 0.09 mmol/l, p = 0.001; 2 h-plasma glucose: 1.22 versus 0.31 mmol/l, p = 0.0002). The percentage of participants with a high level of education (higher vocational education or university) was higher among low-attenders (46.7 %) than among high-attenders (22.4 %)(p = 0.025).

**Table 3:** Attendance at individual and group-consultations of participants in both study groups

**Intervention group**					
**GP consultations**		**NP consultations †**		**Group-consultations**	
**Visit**	**%**	**Visit**	**%**	**Visit**	**%**
Admission	100	baseline	96.8	1 month	71.7
9 months	86.3	3 months	90.6	2 months	63.9
15 months	88.9	6 months	87.1	8 months	58.7
21 months	83.2	12 months	89.0	14 months	50.7
27 months	80.4	18 months	91.2	20 months	38.3
		24 months	86.3		
		30 months	89.1		
**Usual care group**					
**GP consultations**		**NP consultations †**		**Group-consultations**	
**Visit**	**%**	**Visit**	**%**	**Visit**	**%**
Admission	100	baseline	390 (93.7)		
		6 months	353 (86.5)		
		18 months	321 (90.4)		
		30 months	277 (86.0)		

**†** Attendance rates were calculated on all individuals participating in the program at a particular time-point. In total, 479 (intervention) and 446 (usual care) persons started the intervention, of which N = 368 (intervention) and 341 (usual care) individuals completed the programme.

Attendance of intervention-group participants at group-consultations gradually decreased from 72 % to 38 %. Reasons for missing group-consultations were ‘already received enough information from the GP / nurse practitioner’ (24 %), ‘evening doesn’t suit me’ (20 %), and ‘lack of time’ (16 %). No differences in baseline characteristics or clinical outcome measures were observed between participants who attended at least 4 group consultations (80 % or more) compared to participants who did not.

### Attitude of providers

None of the providers in our study declined participation or withdrew from the project. In total, 61 % of the GPs and 83 % of the nurse practitioners were present at all trainings and return-meetings or missed only one meeting. One nurse practitioner (4.2 %) and 15 % of the GPs did not attend any meeting. All nurse practitioners were present at all sessions of the MI-course or missed one session. Eighty-six percent of the providers reported medium or high confidence in diabetes prevention in primary care, while 76 % had medium or high confidence in prevention by lifestyle intervention. Of all providers, 81 % reported medium or high satisfaction with individual counselling.

Twenty-three percent considered the chance of success of diabetes prevention by lifestyle counselling in primary care low or very low. Drop-out was lower and increases in 2 h plasma glucose were smaller among participants receiving counselling from these providers (either GP, nurse practitioner or both) than from providers who considered the chance of success medium or high (drop-out: 5.2 % versus 19.7 % ; p = 0.0017; 2 h plasma glucose: 0.07 mmol/l versus 0.60 mmol/l; p = 0.011). No differences in participant satisfaction with GP or nurse practitioner guidance was found between these two groups of participants.

### Expertise of providers

Nearly all participants that had received advices were satisfied with the level of knowledge of both GPs (94.3 % to 100 %) and nurse practitioners (97.0 to 99.7 %) on each topic discussed. All professionals were confident about their level of knowledge regarding diabetes and weight- and exercise-related topics. For the dietary topics, 80 % of the providers was confident about their basic level of knowledge (role of (saturated) fat and dietary fibre in diabetes prevention, a healthy diet, products high or low in (saturated) fat and dietary fibre).

Half of the participants was satisfied and 40 % was moderately satisfied with the guidance from their GP regarding lifestyle modification. Unsatisfied participants on average had a higher FINDRISC-score (15.8) than moderately satisfied (14.5) or satisfied (14.6) participants (p = 0.003). Satisfied participants more often had a lower level of education (62.9 % no education to lower vocational education) compared to moderately satisfied (41.1 %) or unsatisfied (44.8 %) participants (p = 0.002). No differences in clinical outcomes were found between participants who were (moderately) satisfied or unsatisfied with GP-counselling.

Seventy percent of the participants was satisfied and 25 % was moderately satisfied with the guidance from their nurse practitioner. No differences in baseline characteristics or clinical outcomes were found between participants who were either (moderately) satisfied or unsatisfied with the counselling from their nurse practitioner. All nurse practitioners regarded the MI-course as useful or very useful and all would find such a course desirable or very desirable for nurse practitioners if the programme would be implemented in the Netherlands.

Eighty-five percent of the GPs and all nurse practitioners regarded themselves suitable or moderately suitable for exercise-related guidance. Whereas all nurse practitioners found themselves suitable (63 %) or moderately suitable (37 %) to guide dietary change, nearly 40 % of the GPs found themselves not suitable for nutritional counselling. Another 52 % of the GPs regarded him- or herself moderately suitable. Lack of time and specialistic knowledge (amounts of nutrients in food products, dietary constraints, calculation of calories in the diet) were mentioned by all providers as barriers for guiding dietary change.

### Structure and intensity of the intervention

In total, 75 % of the providers and 86 % of the participants were satisfied with the frequency of the individual consultations and 68 % and 92 % respectively with their duration. Professionals regarded lack of time (60 %), lack of participant motivation for lifestyle change (12 %) and lack of financial reimbursement (11 %) as important barriers for implementation of individual lifestyle counselling in primary care.

Seventy percent of the professionals regarded the GP-practice as the most appropriate setting for group-consultations on lifestyle, as it is ‘a familiar environment for participants that they already relate to their health’. The nurse practitioner, either alone (35 %) or together with the GP (23 %) or a dietician / physiotherapist (21 %) was seen as the key player for organizing such consultations. Lack of practice space (23 %), lack of participant motivation (28 %) and lack of time of professionals (18 %) were mentioned as organisational barriers.

Nearly 90 % of the professionals indicated free-of-charge exercise programmes should be part of lifestyle interventions for diabetes prevention as they ‘offer structured guidance to participants and thereby stimulate motivation’. Of the participants, 54 % was favourable to such programmes and the same percentage would favour personal counselling by a dietician.

## Discussion

Translation of interventions for diabetes prevention into routine clinical practice is challenging [6,7]. We therefore evaluated implementation of a lifestyle intervention for diabetes prevention in Dutch primary care. Based on these insights, we discuss opportunities for refining intervention delivery on the participant, provider and organisational level (Table 4).

**Table 4:** Opportunities for refining intervention delivery on different health care levels

**Level**	**Finding**	**Opportunity for intervention delivery**
**Participant**	* High attendance rates in our study compared to others [11,12,22]	* Use of organisational elements that can contribute to participant compliance:
		*- Immediately plan next appointment during consultations*
		*- Persons who do not show up are contacted by the practice assistant*
		*- Assign 1provider in the practice who is responsible for coordination / planning of the consultations.*
	* Lack of participant motivation experienced by providers as a major barrier for intervention implementation	* Stimulate participant motivation to change unhealthy habits:
		- *In-depth analysis of (barriers for ) participant behavioural change to reveal starting points for refining intervention content*[5,6,23].
		- *More attention for environmental factors promoting unhealthy behaviour*[24]
		- *Counselling based on shared decision making to enlarge participant empowerment*[25]
		- *More effort into stimulating participants to engage social support*[5,23,26].
**Professional**	* Lower participant satisfaction with GP guidance than with nurse practitioner guidance.	* Role for the nurse practitioner as the key player in guiding participant lifestyle change [29,30]
	* Lower self-efficacy of GPs regarding dietary counselling compared to nurse practittioners.	
	* Lack of specialistic nutritional knowledge reported by nurse practitioners	* Introduce elements to fill gaps in knowledge and/or skills of nurse practitioners
	* Nearly 40 % of the nurse practitioners report limited self-efficacy for dietary counselling	- *Referral to skilled supporting staff, like dieticians*[5]
		- *Extend motivational interviewing course towards a specialized prevention manager training*[31], *including modules to enlarge the knowledge of nutrition and physical activity in diabetes prevention.*
**Organisation**	* Lack of counselling time and financial reimbursement regarded by providers as major bottlenecks for intervention implementation	* Consider and investigate prevention strategies that could increase cost-effectiveness [6], such as:
	* Modest diabetes risk reduction compared to studies in experimental settings [8,11,12,26].	- *More stringent criteria for participant inclusion, based on risk*[6,11]*and / or motivation*[27]
		- *Group-counselling*[8-11]
		- *A more tailor-made or patient-centred intervention structure*[6,35]
		- *Integration of lifestyle interventions for different disorders*[36]

### Participant level

In the Netherlands, patients often have a long-lasting relationship with their GP and/or nurse practitioner, whom they consider trustworthy [14]. Individual attention from these providers may therefore lead to high compliance. In line with this hypothesis, attendance at individual consultations was high, which was also found in other studies in general practice [9,11]. Furthermore, drop-out in our study was modest (intervention: 14.6 %; usual care: 13.2 %) and drop-out after 1 year (intervention: 6.3 %; usual care: 7.4 %) was lower than in other prevention studies in primary care (German Praedias-study: 9.3 % [10]; Finnish GOAL-study: 9.4 % [9]). Completers in our study more often had a partner than drop-outs, which was also found in the GOAL study [9].

In our study, no difference in dropout rates between the two study groups was observed. Accounting for the individuals who were lost to follow-up because they developed type 2 diabetes, the statistical power to detect small, but clinically relevant differences in dropout rates between the study groups (Cohen’s conventional effect size of 0.1 [25]) was 0.835. It is therefore unlikely that the lack of a difference in dropout rate between the groups is explained by a lack of statistical power.

In contrast to others [12,13,27], attendance at individual consultations remained high throughout our study, to which several elements in the organisation of our intervention may have contributed. First, following daily routine, appointments for the next visit were made before completing of the current consultation. Second, persons who did not show up at their appointment were contacted by the practice assistant. Third, in each practice, one provider was made responsible for correct implementation of the programme, including coordination of the consultations.

Despite the high compliance in our study, providers regarded lack of participant motivation to change unhealthy habits as an important barrier for effective lifestyle counselling. This result underlines the importance of in-depth evaluation of participant behavioural change in diabetes prevention programmes to reveal starting points for refining intervention content [7], as was for example done by Rosal et al. [6] and Penn et al. [28]. Furthermore, attention should be given to the identification of environmental factors influencing participant behaviour [29], such as the food products offered in worksite cafeterias or the availability of cycling-tracks. As was done in our study, it is recommended that counselling is based on shared decision making to enlarge participant empowerment [30]. Moreover, participants should be stimulated to mobilize social support, which was found to be important for both achieving [6] and maintaining change [27]. In our study, partner support was also found to contribute to weight loss success [23].

### Professional level

A positive attitude of health care providers towards change is indispensable for implementation of innovations in clinical practice [8]. Satisfying this condition, the majority of providers in our study was confident about diabetes prevention by lifestyle counselling in Dutch primary care. Furthermore, attendance at training sessions was high and none of the providers refused participation or withdrew from the study. It must be remarked that provider compliance may be overestimated in our study as all practices were part of an association, which as a whole decided to participate in the project. In two recent Dutch studies [16,31], GPs were however also found to attach high importance to chronic disease prevention.

Remarkably, drop-out was lower and increases in 2 h plasma glucose were smaller in participants receiving counselling from providers who considered the chance of success of diabetes prevention in Dutch primary care low or very low than in participants from providers who considered the chance of success medium or high. In line with these results, it was previously found in our study that a lack of motivation or confidence of providers does not negatively influence participant guidance [26]. These results may reflect a professional attitude, in which personal barriers to diabetes prevention by lifestyle intervention do not affect participant counselling [26]. Furthermore, a reserved attitude of providers towards prevention obviously does not imply a lack of capacity for guiding lifestyle change.

Although primary care is regarded as a highly suitable setting for disease prevention [32], debate is ongoing about the optimal division of the workload between the GP and the nurse practitioner [33,34]. The lower participant satisfaction with GP guidance and the lower self-efficacy of GPs regarding dietary counselling in our study support a role for the nurse practitioner as the key player in guiding lifestyle change. As most nurse practitioners in the Netherlands provide care to diabetes patients [16], this role is compatible with existing routines. Moreover, Dutch GPs recently reported a preference for nurse practitioners to perform preventive activities [16]. In our study the MI-course was however only offered to nurse practitioners, which may have contributed to their skills. Furthermore, after the admission interview, participants did not meet with the GP for nine months, which may have influenced their perception of GP importance in the study.

Although our findings suggest that they are very suitable to guide participant lifestyle change, nurse practitioners in our study reported a lack of nutritional knowledge. Moreover, nearly 40 % regarded themselves as only moderately suitable for dietary counselling. Referral to dieticians may be necessary to fill these gaps and may furthermore relieve time–pressure for nurse practitioners [6,16]. As an alternative, the motivational interviewing course –which was considered useful and desirable by all nurse practitioners in our study- may be extended towards a specialized prevention manager training [35], which also focuses on aspects of nutrition and physical activity in diabetes prevention. This training could then for example only be offered to a subset of highly motivated nurses, to which all GPs in a certain region can refer [36]. A disadvantage of this latter approach is however that participants may not receive counselling from their familiar provider and/or in their own practice.

### Organisational level

Most providers in our study were satisfied with both the frequency and the duration of the individual consultations. However, comparable to other studies [6,7], limited counselling time was regarded as an important bottleneck for programme implementation. Furthermore, although individual lifestyle interventions can save money even when effectiveness is low [37], financial reimbursement for preventive activities is mostly lacking [6,31]. Moreover, in several programmes in clinical practice –including ours-, risk factor reductions were modest compared to studies in experimental settings [9,12,13,30]. In diabetes translational research, it is therefore essential to consider strategies that could increase cost-effectiveness [7].

A first approach could be to allow less persons to participate by applying more strict ‘selection at the gates’. Following other programmes [9,11,38], a FINDRISC value of 13 points was chosen in our study as selection criterium, which may have led to inclusion of individuals with a relatively healthy lifestyle. Furthermore, disturbed glucose values were not a prerequisite for participation. More stringent criteria -and thus a less favourable risk profile- leave more room for improvement and may lead to higher participant efforts [7,12]. In addition, pre-screening based on the motivation to change the lifestyle, –as was done in the Dutch ‘beweegkuur’ [31]- may be useful to include only those most willing to change.

A second strategy to reduce costs and thus potentially increase cost-effectiveness is group-counselling, which was applied in several prevention programmes [9-12]. However, although most providers in our study regarded primary care as an appropriate setting for group-based lifestyle interventions, attendance at group-consultations was low. This could be explained by the purely didactive nature of the group-meetings and by the fact that they were supplementary to individual counselling. In line with this hypothesis, ‘I already received enough information from the GP / nurse practitioner’ was on often-mentioned reason for missing group-meetings. In other studies however, participants also reported a preference for personal guidance [31,39]. Furthermore, in several group-based interventions the number of participants initially refusing to participate was not mentioned [9-11]. Preceding analysis of the attractiveness of group-counselling for participants is therefore necessary.

Third, a more tailor-made counselling approach may be considered [7,40]. Although regarded useful and desirable by most providers, only half of the participants in our study for example favoured exercise programmes and personal dietary counselling. A better adapted or more patient-centred intervention structure may result in higher participant compliance. Furthermore, restricted offering of intervention modules may reduce costs. Based on their preferences, persons may for example participate in (a combination of) weight loss, dietary and exercise modules, offered by means of brief, intensive or group-based counselling, whether or not supported by exercise programmes or dietary guidance. However, feasilibity, acceptability and (cost-)effectiveness of such a design require further research.

Fourth, as the risks of an unhealthy lifestyle are not confined to diabetes, individuals may be enrolled in several behavioural change initiatives at the same time, which is time- and money-consuming. Integration of screening [41] and/or intervention [31] for different disorders is therefore recommendable, whereby general intervention modules aimed at shared risk factors may be supplemented with disease specific components.

### Strengths and limitations

In our study we evaluated a wide spectrum of opportunities for diabetes prevention in Dutch primary care. The high questionnaire response rates make it unlikely that missing values have significantly influenced the results. However, when filling out the questionnaires, both participants and professionals may have been affected by recent experiences. In addition, the missing records from drop-outs and individuals with diabetes may have influenced participant outcomes. Reasons for withdrawal did not however indicate dissatisfaction among drop-outs. Self-reported outcomes of participants and providers on knowledge and skills of professionals must be interpreted with caution.

## Conclusions

High participant compliance and a positive attitude of providers make primary care a suitable setting for diabetes prevention by lifestyle counselling in the Netherlands. Results support a role for the nurse practitioner as the key player in guiding lifestyle modification, whereby referral to supporting staff or more extended training may be necessary. Further research is needed on participant behavioural change and on strategies that could increase cost-effectiveness, such as more stringent criteria for participant inclusion, group-counselling, more tailor-made counselling and integration of interventions for different disorders.

## Competing interests

All authors declare that they have no competing interests.

## Authors’ contributions

PV contributed to the study design, to the acquisition of the data, and to analysis and interpretation of the data and drafted the manuscript. IM, FW, CB, JS, GW and HvO contributed to the study design and to the interpretation of the data and critically revised the manuscript. All authors read and approved the final manuscript.

## Pre-publication history

The pre-publication history for this paper can be accessed here:

http://www.biomedcentral.com/1471-2296/13/79/prepub
